# On the Causes of Death of Animals Subjected to High Temperatures

**Published:** 1860-06

**Authors:** 


*On the Causes of Death of Animals subjected to High Temperatures.*—M. Claude Bernard states that warmblooded vertebrate animals, inclosed in a dry and heated atmosphere live for a certain time without any appreciable effect being indicated, except accelerated respiration and circulation, death occurring suddenly after the lapse of some time, which varies with the elevation of temperature, the size of the animal, its age, etc. The cause of death in these cases, he believes to be attributable solely to the elevation of temperature of the blood, independently of other circumstances.— In the mammifera and birds, the temperature may be raised from four to five degrees C. above the normal condition, the animals infallibly perishing whenever this limit is exceeded, and at the moment of death the heart being found rigid along with the other muscles of the body. The interesting conclusion drawn from his experiments is, that a purely physical condition of the blood—mere increase of its temperature—may be a cause of death. Another point also interesting in connection with this circumstance is, that the amount of increase in temperature sufficient to produce this effect is well marked and invariable, being, from four to five degrees C. above the normal standard in warm-blooded animals—that is, between forty-five and forty-six degress C. in mammifers, and fifty-one and fifty-two degrees C. in birds. This M. Bernard explains as arising from the fact that such increase of temperature is incompatible with the exercise of muscular contractility, and consequently leads to arrestment of the heart's action. M. Kuhe has shown that in such cases the rigidity is due to the coagulation of a certain matter contained in muscular tissue.—*Gaz. Medicale*, July 1859, *Edin. Med. Jour.*, Dec., 1859.

